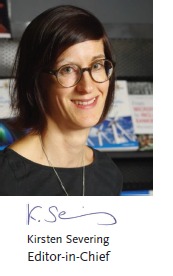# *Advanced Science*–Growing Further and Further

**DOI:** 10.1002/advs.201802184

**Published:** 2019-01-09

**Authors:** Kirsten Severing

**Figure 1:**
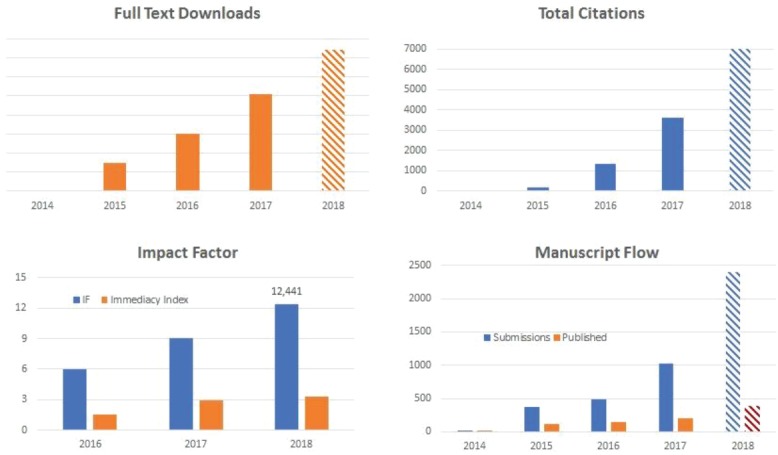
Selected key performance indicators.

Welcome to the 6^th^ volume of *Advanced Science*. Like in previous years, there is so much positive news to spread for *Advanced Science*. All key performance indicators that characterize a flourishing journal show a very positive trend (**Figure** **1**). Our content is popular like never before–the number of full text downloads increased by another 40% in 2018. Looking at the number of total citations in 2018 makes us optimistic that we will see another increase in the impact factor in 2019. The number of submissions had already been high and growing steadily during the first half of the year 2018. After the announcement of the new impact factor of 12.441 (+40%), we saw another big jump in the number of papers submitted. We continue to select the best papers following our strict and rigorous peer‐review process. Still, the number of published articles is growing as well. While the submissions have more than doubled in 2018, we published about 60% more compared to 2017. This year we will therefore publish biweekly issues.

But it is not only an increase in quantity that we have experienced. The variety of topics has broadened as well: initially dominated by materials science research, other communities have now discovered *Advanced Science* as an attractive publishing platform. In order to additionally promote this content and help guide interested readers to the bio and life science‐oriented content, we recently launched our **Health, Medical, and Life Sciences Virtual Issue**. Updated on a regular basis, this ongoing series contains an editorial choice of our favorite articles in areas like CRISPR, cancer therapy, synthetic biology, and antibacterial hydrogels. Maybe next year's top cited papers (**Table** **1**) will contain more of these topics as well.

**Table 1:** Most cited papers in 2018

Title	Corresponding Author	Publication Date	Total	2018
A Review of Solid Electrolyte Interphases on Lithium Metal Anode	Qiang Zhang et al., Tsinghua University, Beijing, China	March 2016	301	153
Transition Metal Carbides and Nitrides in Energy Storage and Conversion	Hong Jin Fan, Nanyang Technology University, Singapore	May 2016	207	79
Advances in Perovskite Solar Cells	David Cahen, Liming Ding et al., Weizmann Institute of Science, Israel and National Center of Nanoscience & Technology, Beijing, China	July 2016	161	75
Janus Separator of Polypropylene‐Supported Cellular Graphene Framework for Sulfur Cathodes with High Utilization in Lithium‐Sulfur Batteries	Qiang Zhang et al., Tsinghua University, Beijing, China	January 2016	141	47
Battery‐Supercapacitor Hybrid Devices: Recent Progress and Future Prospects	Jinping Liu, Jianlong Xia et al., Huazhong University of Science and Technology, Wuhan and Wuhan University of Technology, China	July 2017	110	87
One‐dimensional TiO2 Nanotube Photocatalysts for Solar Water Splitting	Yuekun Lai et al., Soochow University, Suzhou, China	January 2017	98	64
Advanced Micro/Nanostructures for Lithium Metal Anodes	Qiang Zhang, Yu‐Guo Guo et al., Tsinghua University and Institute of Chemistry, Chinese Academy of Sciences, Beijing, China	March 2017	94	66
Progress and Challenges in Transfer of Large‐Area Graphene Films	Jing‐Gang Gai et al., Sichuan University, Chengdu, China	August 2016	90	56
Z‐Scheme Photocatalytic Systems for Promoting Photocatalytic Performance: Recent Progress and Future Challenges	Zhigang Zou, Yong Zhou et al., Nanjing University, China	November 2016	91	62
Paper‐Based Electrodes for Flexible Energy Storage Devices	Yat Li et al., University of California, Santa Cruz, CA, USA	July 2017	88	71

**Figure 2:**
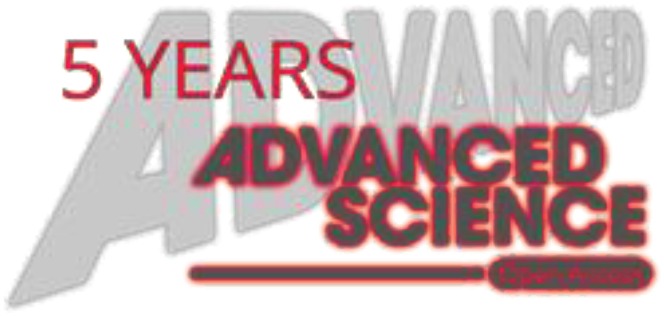
Anniversary logo.

On the occasion of the journal's 5^th^ anniversary that is approaching in December of this year, we have started a **celebratory series** of invited‐only articles from our executive advisory board members, providing both an overview of progress, and, importantly, insight into possible future directions of their respective fields (**Figure** **2**). Also, this series will be updated regularly as new 5^th^ anniversary articles are published. We are positive that these articles will become standard reading and inspire the next generation of scientists.

With this, I would like to thank all of you, editorial board members, authors, reviewers, and readers, for your continued interest in *Advanced Science.*

I hope you will follow us on our journey into the next exciting year.

On behalf of the editorial team,